# Ultrasound Localization Microscopy for the Assessment of Microvascular Circulation in Ischemic Perinatal Stroke

**DOI:** 10.1161/STROKEAHA.124.048390

**Published:** 2024-10-30

**Authors:** Adrian P. Regensburger, Felix Wachter, Louise Denis, Henriette Mandelbaum, Frauke Schey, Adrian Buehler, Gregor Siebenlist, Simone Schwarz, Susanne Schulz-Heise, Oliver Rompel, Regina Trollmann, Joachim Woelfle, Jörg Jüngert, Olivier Couture, Heiko Reutter, Gregor Hanslik, Ferdinand Knieling

**Affiliations:** Department of Pediatrics and Adolescent Medicine (A.P.R., F.W., H.M., F.S., A.B., G.S., R.T., J.W., J.J., H.R., G.H., F.K.), University Hospital Erlangen, Germany.; Institute of Radiology (S.S.-H., O.R.), University Hospital Erlangen, Germany.; Laboratoire d’Imagerie Biomédicale, Sorbonne Université, Centre National de la Recherche (CNRS), Institut National de la Santé et de la Recherche Médicale (INSERM), Paris, France (L.D., O.C.).; Department of Neonatology and Pediatric Intensive Care Medicine, Sana Clinics Duisburg, Germany (S.S.).

**Keywords:** diagnostic imaging, microcirculation, neonate, stroke, ultrasound

In pediatrics, newborns are at the highest risk for an ischemic stroke with an incidence of 1 in 3500 in live births and subsequent development of neurological deficits.^1^ While the onset of symptoms is commonly on the first day of life, diagnosis by cerebral imaging, with magnetic resonance imaging as the reference standard, is often delayed.^2^ That is why the American Heart/Stroke Association has called for the development of novel bedside clinical assessment methods.^1^ Off-label intravenous application of contrast (sulfur hexafluoride microbubbles) for transfontanellar contrast-enhanced ultrasound (TCEUS) might overcome these limitations,^3^ and by advanced algorithms for tracking individual microbubbles, super-resolution imaging of microvascular networks is enabled (ultrasound localization microscopy [ULM]).^4^ Herein, we present the first application of ULM in a case of perinatal ischemic stroke.

A late-term newborn (41+5, 3625-g birthweight) with good postpartum adaption underwent multimodal neuroimaging after presenting with recurrent apnea and focal seizures on the third day of life in an external emergency department. Here, electroencephalography revealed abnormal left-sided focal spikes, cerebral edema in ultrasound, and acute ischemia in the left middle cerebral artery area in head computed tomography. On the fifth day of life, head magnetic resonance imaging confirmed the diagnosis of an acute ischemic stroke in the middle cerebral artery (M2 and M3) with involvement of the insula and basal ganglia. Bedside TCEUS on the same day and time-intensity curves analysis revealed the same ischemic area and further residual perfusion in the stroke region and hyperperfusion of the remaining areas of the left hemisphere. In the postacute phase, 17 days after the first magnetic resonance imaging/TCEUS, reestablishment of blood circulation at a lower level in the ischemic area and a reduction in hyperperfusion could be observed by TCEUS with respective time-intensity curves analysis (Figure [A and B]; Video S1). Further standard diagnostics could not find a clear cause for the stroke.

**Figure.**
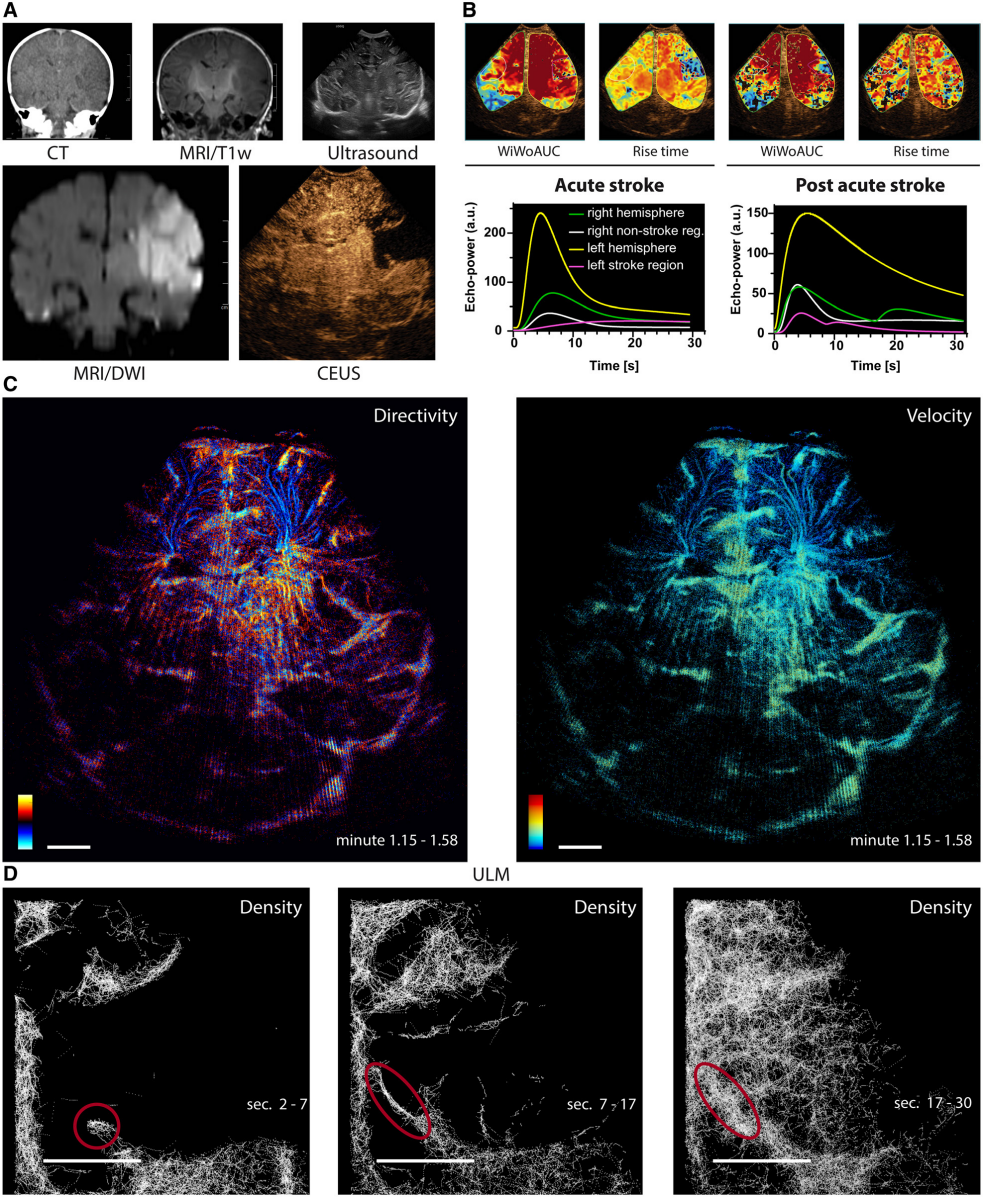
**Ultrasound localization microscopy (ULM) of perinatal ischemic stroke. A**, Frontal plane images of the head of a neonate by computed tomography (CT), magnetic resonance imaging/diffusion-weighted imaging (MRI/DWI; reconstructed), and B-mode ultrasound. Left middle cerebral artery infarction was detected by all imaging modalities. Diffusion-weighted MRI sequence and transfontanellar contrast-enhanced ultrasound (CEUS) were superior for diagnosing the stroke region. **B**, Time-intensity curve analyses of cerebral perfusion of the left and right hemispheres, the ischemic area, and the respective area on the opposite side. Visualization of 2 calculated perfusion parameters (area under curve during wash-in and wash-out phase [WiWoAUC] and the rise time) within the regions of interest. Imaging was performed during the acute and postacute phases unveiling perfusion improvement in the infarcted area. **C** and **D**, ULM of the whole brain (**C**) and time-resolved of the infarcted area (**D**) during the acute phase. Displayed are directivity and velocity maps during the low flow phase (1.15–1.58 minutes after the first bubble arrival) showing hyperperfusion of the left hemisphere. The stroke area was further analyzed directly after the first microbubble arrival and the consecutive time periods (2–7, 7–17, and 17–30 seconds) to demonstrate the residual vascularity in the stroke area by time (circles). Scale bar, 1 cm.

ULM (Figure [C and D]) reconstructions were individually adjusted based on a code publicly available at https://github.com/AChavignon/PALA.^5^ Microbubbles were enhanced with a spatiotemporal filter and tracked with the Hungarian algorithm.^6^ The individually tracked microbubbles were then quantified for directivity, velocity, and density. The ULM density map shows the number of microbubbles tracked per pixel, which corresponds to the blood flow within the reconstructed vessels (Figure [D]; Video S2). In the infarcted region during the acute phase, decreased density of microbubbles, that is, vessel density, in comparison to the unaffected side was detected. However, we identified specific microvessels with residual blood circulation over time in the stroke area with increasing number of bubbles (density) from 0.8×10^−3^ to 15.8×10^−3^ and 70.6×10^−3^, while it remained stable in the control region. Furthermore, the velocity of the left compared with the right cortex and hemisphere was accelerated from 2.08 to 2.33 and 1.72 to 2.29 mm/s, respectively, as a sign of left-sided cerebral hyperperfusion.

We demonstrated the potential of TCEUS for bedside diagnosis and the first application of ULM to provide super-resolution and time-resolved information on the microvascular structure and circulation in perinatal stroke. Due to the wide availability and immediate bedside and safe^3^ applicability of TCEUS/ULM in newborns with seizures, this might accelerate the diagnosis of acute perinatal stroke and, thus, influence clinical decisions and may even be used for early therapeutic approaches in the future. This should be further investigated in clinical studies.

## Article Information

### Acknowledgments

The authors thank the family for giving their written consent to the publication of the case.

### Sources of Funding

This work was supported by Else Kröner-Fresenius-Stiftung (Else Kröner Excellence Fellowship to Dr Regensburger), the European Research Council Starting grant (101115742-IseeG to Dr Knieling), and the European Research Council Consolidator grant (772786-ResolveStroke to Dr Couture).

### Disclosures

Dr Couture is a co-inventor of ultrasound super-resolution patent (Patent Cooperation Treaty [PCT])/FR2011/052810 and a co-founder of Resolve Stroke and receives compensation from Bracco Imaging for consultant services. Dr Knieling reports grants from the H2020 European Research Council, Bayerische Forschungsstiftung, Sanofi-Aventis, and Siemens Healthcare and receives compensation from iThera Medical GmbH for consultant services and travel support from Sanofi-Aventis. The other authors report no conflicts.

### Supplemental Material

Videos S1–S2

## Supplementary Material


